# Environmental Surveillance for Poliovirus and Other Enteroviruses: Long-Term Experience in Moscow, Russian Federation, 2004–2017

**DOI:** 10.3390/v11050424

**Published:** 2019-05-08

**Authors:** Olga E. Ivanova, Maria S. Yarmolskaya, Tatiana P. Eremeeva, Galina M. Babkina, Olga Y. Baykova, Lyudmila V. Akhmadishina, Alexandr Y. Krasota, Liubov I. Kozlovskaya, Alexander N. Lukashev

**Affiliations:** 1 Federal State Budgetary Scientific Institution “Chumakov Federal Scientific Centre for Research and Development of Immune-and-Biological Products of the Russian Academy of Sciences” (FSBSI “Chumakov FSC R&D IBP RAS”), 108819 Moscow, Russia; poliom_ldms@mail.ru (T.P.E.); baykovaaa@mail.ru (O.Y.B.); krasota@belozersky.msu.ru (A.Y.K.); lubov_i_k@mail.ru (L.I.K.); 2 Sechenov First Moscow State Medical University, 119991 Moscow, Russia; akhludmilav@gmail.com; 3 Federal Budget Institution of Health of Rospotrebnadzor “Center for Hygiene and Epidemiology in Moscow”, Moscow 129626, Russia; mashay@bk.ru (M.S.Y.); poliolab@yandex.ru (G.M.B.); 4 Belozersky Institute of Physical-Chemical Biology, Lomonosov Moscow State University, 119899 Moscow, Russia

**Keywords:** enterovirus, poliovirus, sewage, environmental surveillance, enterovirus surveillance

## Abstract

Polio and enterovirus surveillance may include a number of approaches, including incidence-based observation, a sentinel physician system, environmental monitoring and acute flaccid paralysis (AFP) surveillance. The relative value of these methods is widely debated. Here we summarized the results of 14 years of environmental surveillance at four sewage treatment plants of various capacities in Moscow, Russia. A total of 5450 samples were screened, yielding 1089 (20.0%) positive samples. There were 1168 viruses isolated including types 1–3 polioviruses (43%) and 29 different types of non-polio enteroviruses (51%). Despite using the same methodology, a significant variation in detection rates was observed between the treatment plants and within the same facility over time. The number of poliovirus isolates obtained from sewage was roughly 60 times higher than from AFP surveillance over the same time frame. All except one poliovirus isolate were Sabin-like polioviruses. The one isolate was vaccine-derived poliovirus type 2 with 17.6% difference from the corresponding Sabin strain, suggesting long-term circulation outside the scope of the surveillance. For some non-polio enterovirus types (e.g., Echovirus 6) there was a good correlation between detection in sewage and incidence of clinical cases in a given year, while other types (e.g., Echovirus 30) could cause large outbreaks and be almost absent in sewage samples. Therefore, sewage monitoring can be an important part of enterovirus surveillance, but cannot substitute other approaches.

## 1. Introduction

Human enteroviruses are small non-enveloped RNA viruses, which belong to species enterovirus A, B, C, and D (genus *Enterovirus*, family *Picornaviridae*) and include viruses historically designated as coxsackieviruses A (CVA), coxsackieviruses B (CVB), and echoviruses (E). Polioviruses types 1–3 belong to the enterovirus C species. Enteroviruses (EVs) are ubiquitous and are spread via fecal–oral or aerosol routes, and in the vast majority of infected people, the infection is asymptomatic. Only a small fraction of infected individuals have clinical manifestations, which range from mild intestinal or catarrhal symptoms to severe lesions of the nervous system, such as meningitis, encephalitis, and poliomyelitis. EVs are therefore regularly excreted into the environment with human feces and contaminate wastewater and other objects. Moreover, since the introduction of a live oral poliovirus vaccine made of Sabin strains (OPV) in the late 1950s, Sabin vaccine strains regularly enter sewage globally. EVs are relatively resistant to environmental factors, such as temperature and pH, and may remain infectious for a long time. Thus, monitoring of EVs in wastewater provides data on the EV circulation in the population, including asymptomatic infections.

In the context of the Global Polio Eradication Initiative, wastewater monitoring has acquired a particular importance, especially at the final stage of the program, and will no doubt be an important part of poliovirus surveillance after global certification [1,2,3]. Environmental surveillance, which is largely constituted by sewage monitoring, allows the identification of “silent” circulation or introduction of wild and vaccine-derived polioviruses into the population in the absence of clinical cases of poliomyelitis [4,5,6,7,8,9], which is extremely important for epidemiological assessment and timely response [10].

In Russia, virological studies of wastewater were carried out from the middle of the 20^th^ century to assess the quality of wastewater treatment and obtain information on circulating EVs. These studies were optional; they did not necessarily use a common methodology. In 1996, Russia adopted the National Poliomyelitis Eradication Program and introduced acute flaccid paralysis (AFP) surveillance and wastewater monitoring as a mandatory component of the poliovirus surveillance. Since 2008, Russia has implemented a national program called “Epidemiological surveillance and prophylaxis of non-polio enterovirus infection”, which also recommends wastewater surveillance to monitor the circulation of non-polio enteroviruses (NPEVs). The methodology of wastewater monitoring in Russia is based on the recommendations of the World Health Organization (WHO) [11] and national guidelines [12] aimed to primarily identify polioviruses.

This report presents the results of a virological study of wastewater in Moscow for over 14 years, 2004–2017, in relation to AFP surveillance and clinical registration of enterovirus infections.

## 2. Materials and Methods

### 2.1. Sample Collection and Concentration

**Figure 1:**
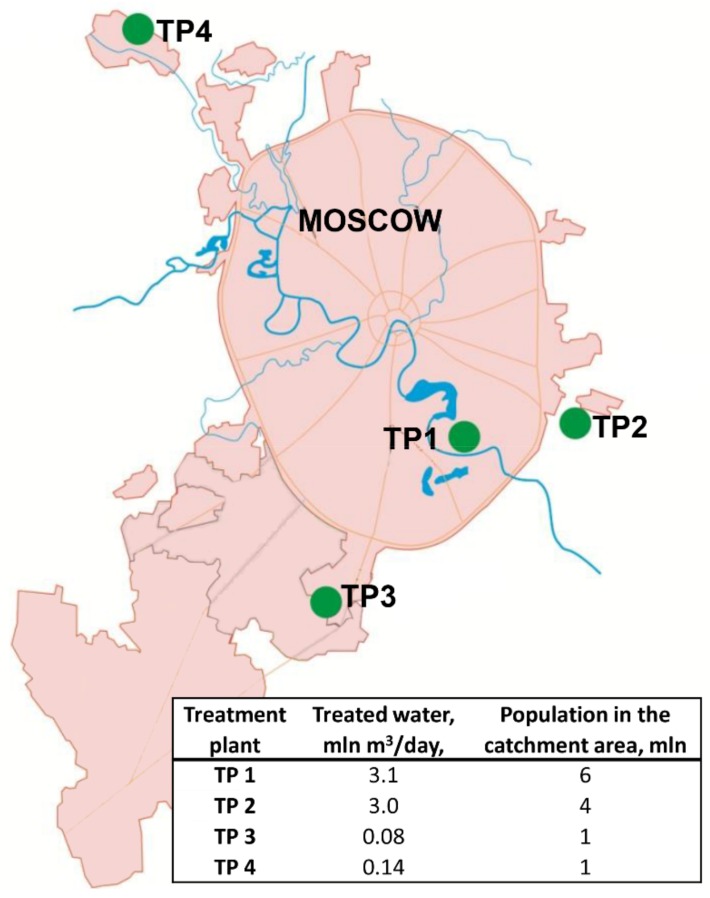
Location, throughput, and population in the catchment area of Moscow sewage treatment plants (TP).

Moscow is the capital and the largest city of Russia with an area of 2561 km^2^, and a population of 12,506,468 people, located in a zone of temperate continental climate with a pronounced seasonality. Nine major railway stations, four airports, and three river ports are situated in Moscow. About 1.5–2 million people arrive in Moscow every day from adjacent regions as daily migration. The municipal sewage system collects domestic and industrial wastewater, street drains, etc., into the same system. Then this combined wastewater undergoes a full treatment cycle at four sewage treatment plants (TP) (Figure 1).

Samples of wastewater were collected weekly at the stage after mechanical sewage treatment by trap sampling using bags with sorbent (macroporous glass) [11], which were exposed in the sewage stream for 7 days. Afterwards, the sorbent from the bag was transferred into a 10 mL glass column. For stepwise elution three solutions (3.0 mL each) were used: 0.05 M Tris-HCl pH 9.1, 0.05 M Tris-HCl pH 9.1 with 0.5 M NaCl, 3% beef extract in 0.05 M Tris-HCl pH 9.1. Three eluates (3.0 mL each) were treated with chloroform [13] and used for virus isolation.

### 2.2. Virus Isolation

Each chloroform-treated eluate or stool sample was inoculated in three 25 cm^2^ flasks with L20B, Hep-2 (Cincinnati) and RD cells in accordance with the WHO operating procedures [13]. Flasks were incubated for 7 days at 36 °C. Two serial blind passages were carried out in all cell lines. Cell cultures without signs of cytopathic effect were assumed negative. Samples with cytopathic effect on RD cells were additionally passaged on L20B cells to identify polioviruses.

### 2.3. Virus Identification and Characterization

Virus identification was performed in a neutralization assay according to the standard WHO protocol [13] with polyclonal sera (RIVM, Bilthoven, the Netherlands) for identification of poliovirus type 1–3 and pools A–G for identification of 20 EV-B types and parechoviruses. Intratypic differentiation of polioviruses was carried out using a direct ELISA [14], RT-PCR and Real-time RT-PCR [15,16]. Isolates exhibiting equivocal properties in intratyping differentiation methods were sequenced. For that purpose, isolation of total RNA from the suspension of infected cells, reverse transcription, PCR amplification of the poliovirus genome fragments encoding the VP1 protein, their purification and sequencing were done as described [17]. Most of the non-polio enteroviruses that could not be identified in the neutralization test (non-typed viruses, NTVs) were typed by partial VP1 sequencing as described previously [18].

### 2.4. Statistical Methods

The reliability of comparing the results was evaluated by Mann–Whitney U test as described by Gubler [19] using the OriginPro 8.0 software (OriginLab Corporation).

## 3. Results

**Figure 2:**
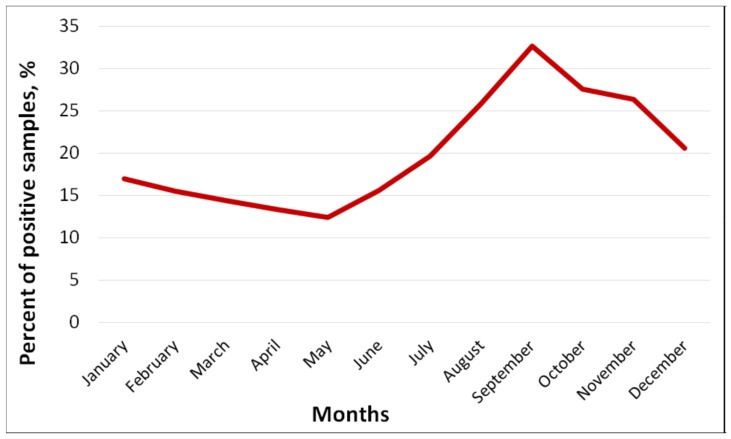
Seasonal frequency of isolation of enteroviruses (%) from sewage, Moscow, 2004–2017.

Within 14 years, 5450 sewage samples were collected at all four TPs, and 1089 samples (20.0%) were positive for EVs (Table 1). The total proportion of positive samples ranged from 8.9% in 2016 to 26.6% in 2012. The frequency of EV isolation at individual TPs over the entire observation period ranged from 14.4% to 34.6% (Table 1). It was the highest (*p* < 0.05) at the smaller capacity sewage treatment plants: 34.6% at TP 3 and 32.5% at TP 4. The number of sewage samples containing different EVs was the highest during the summer–autumn period (August–November) with a maximum in September (32.7%) (Figure 2).

**Figure 3:**
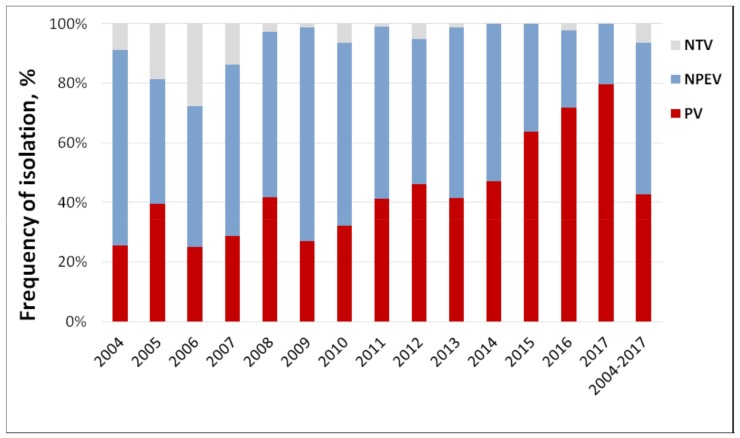
Annual ratio of polio and non-polio enteroviruses in sewage samples, Moscow, 2004–2017. NTV—non-typed virus, NPEV—non-polio enterovirus, PV—poliovirus.

Over the entire observation period (2004–2017), 1168 viruses were isolated from wastewater (79 samples contained multiple viruses), of which 42.7% were polioviruses (PVs), 50.9% were different NPEVs, and 6.3% could not be identified (NTV, Figure 3), but are likely enteroviruses. The proportion of NPEVs was the highest in 2009 (71.8%) and the lowest in 2017 (20.4%); the proportion of PVs was the highest in 2017 (79.6%) and the lowest in 2006 (25%). The largest number of isolates that were not identified was in 2006 (27.6%). This can be attributed to an unestablished methodology after the introduction of monitoring. Also, the isolation protocol was primarily targeted at resolving EV/PV mixtures, while some of the non-typed viruses could be mixtures of non-polio enteroviruses.

**Figure 4:**
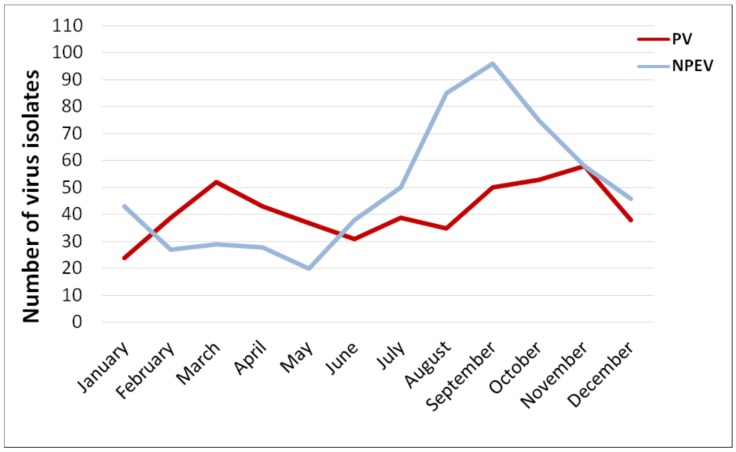
Average monthly isolation of polio and non-polio enteroviruses in sewage samples, Moscow, 2004–2017. NTVs are not included in the NPEV count.

The monthly rate of PV isolation was almost the same throughout the year, with a slight increase in March and November. It could be linked to fewer holidays and vacations (and thus more OPV administrations) in February–April and September–November; however, monthly data on IPV administration to support this speculation is lacking. The rate of NPEVs isolation had a pronounced summer–autumn peak (July–October) with a maximum in September (Figure 4).

During the period of trivalent OPV use (2004–April 2016) type 3 was the most frequent poliovirus (178 isolates, 45%), followed by PV type 2 (144 isolates, 36.5%) and type 1 (73 isolates, 18.5%). After the switch to bivalent OPV type 3 was also the most common, 77 of 104 isolates (74%). The last isolation of PV type 2 was in January 2016, three months before the switch. After that, no PV type 2 was isolated from the wastewater.

All PV isolates were of vaccine origin, all but one, could be classified as vaccine-like. In a sewage sample collected in October 1, 2015 at TP1, a highly divergent vaccine-derived poliovirus (VDPV) type 2 was isolated. The isolate differed from the prototype Sabin 2 strain by 17.6% of nucleotide sequence in the VP1 genome region. This is compatible with about two decades of circulation, assuming 1% per year substitution rate [20] or a comparably long persistence in an immunocompromised individual [21].

Among the NPEVs (Table 2), the largest number (556 isolates, 93.5%) belonged to the EV-B species. The most frequently isolated types were E7 (25.7%), E11 (19%), E6 (11.8%), viruses of the CVB 1–6 group (11.1%, of them CVB5 was the most common and comprised 37.9%), E3 (7.1%), and E19 (4.2%).

**Figure 5:**
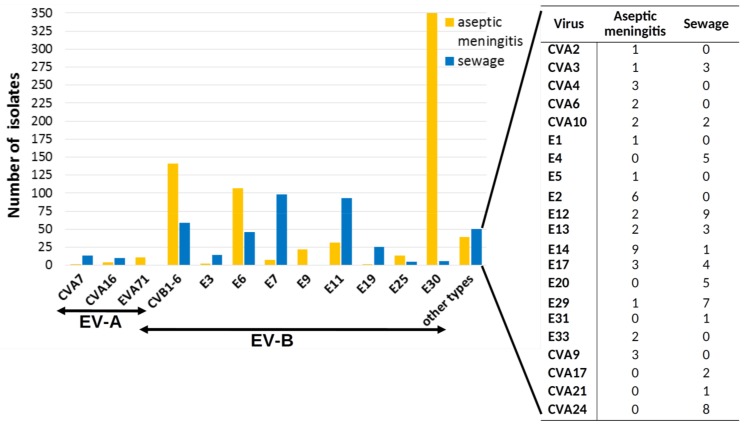
Spectrum of NPEV isolated from sewage and from cases of aseptic meningitis, Moscow, 2008–2017.

To assess the prognostic potential of the sewage investigation in relation to enterovirus infections, we compared the repertoire of NPEVs isolated from wastewater with the spectrum of NPEVs isolated from stool samples of patients with aseptic meningitis (AM) registered in Moscow in 2008–2017. Since 2008, AM is subject to mandatory registration and laboratory investigation in Russia. Samples of feces were collected and tested in RD, Hep-2, and L20B cell lines in accordance with the officially approved guidelines [22]. The spectrum of NPEV isolated from AM cases in 2008–2017 (29 types) slightly exceeded the spectrum of NPEVs isolated from wastewater (23 types) over the same time frame (Figure 5). Only two echovirus types (E6 and E11) and CVB 1–6 were highly prevalent both among meningitis patients and in wastewater. The E7 (the leading type in the sewage) and E19 were rarely isolated from AM cases. Moreover, E30, which was the leading causative agent of AM, was practically absent in wastewater.

**Figure 6:**
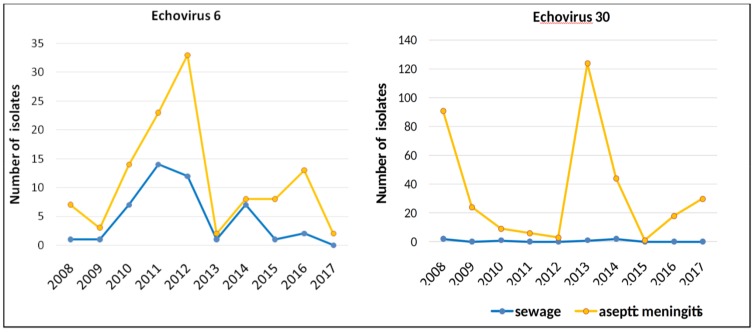
The dynamics of isolation of E6 and E30 from sewage and from cases of aseptic meningitis in Moscow, 2008–2017, by year.

Comparison of the annual isolation dynamics of two most common AM causative agents, E6 and E30, from sewage and AM cases (Figure 6) indicates that the dynamics of sewage isolation coincided with the dynamics of AM cases isolation for E6, but not for E30. For over 10 years, almost no E30 specimen was isolated from wastewater.

## 4. Discussion

### 4.1. Sewage Monitoring in Poliovirus Surveillance

The choice of the optimal poliovirus surveillance strategy is difficult. It depends upon many factors, including the goals and the available means [2]. For the purposes of polio eradication strategy, AFP surveillance remains a gold standard. Theoretically, it is capable of detecting a single case of paralytic poliomyelitis or about one of several hundred infections by an emerging neurovirulent poliovirus, assuming 1:200 rate of clinical to asymptomatic poliomyelitis [23]. However, in the scenario of a real wild PV outbreak in Israel in 2013, it was assumed to have a sensitivity of 1 out of 7000 poliovirus infections [24], and in the inactivated polio vaccine (IPV)-immunized population it might have even lower sensitivity. AFP surveillance also provides an overview of the circulating NPEV landscape. On the other hand, the cytopathic types, such as all EV-B and most EV-A types, are over-represented if cell culture detection is used, which is a standard procedure recommended by the WHO. Initially it was considered that for the purposes of the polio eradication program, data obtained from AFP surveillance will be sufficient. However, the accumulating evidence of the “silent” circulation of polioviruses (i.e., the isolation of polioviruses from wastewater in the absence of manifest cases) made environmental surveillance an important additional part of poliovirus surveillance. It is attractive because it can be easily organized and standardized, does not depend upon clinical reporting, and is more cost-efficient for circulating virus monitoring. We have shown here that the sewage monitoring can yield very useful data, but also has serious shortcomings.

A highly divergent VDPV type 2 has been detected by the sewage monitoring in Moscow among a total of 499 PV isolates. This virus differed from the corresponding Sabin strain by over 17% nucleotide sequence of VP1. Taking into account that poliovirus accumulates about 1% substitutions in VP1 per year [19], it is most likely that this virus was excreted by an immunodeficient vaccine recipient (or a contact of an OPV recipient), since such a long-term circulation of VDPV in a highly immunized population [25] is unlikely, although it cannot be completely excluded. It is noteworthy that a targeted search for persisting polioviruses in immunocompromised children, which are a risk group for preserving divergent viruses, was carried out in Russia, including Moscow, in 2008–2013 (136 enrolled patients) [26] and in 2014–2015 (83 enrolled patients) [27]. No persisting polioviruses were detected therein. Therefore, even well-organized and sensitive patient-targeted surveillance can miss a certain number of cases, and environmental monitoring is indispensable for polio surveillance. In our case, although this virus could have been imported from another region, the sewage monitoring has surely proven its value.

Another important result was the absence of PV2 in sewage after cessation of type 2 OPV. It has been shown that even in absence of OPV, a substantial fraction of sewage samples (up to 8%) can contain Sabin 2 strains [28]. No type 2 Sabin strains were found among 673 sewage probes collected over 20 months after OPV2 withdrawal, while 27 Sabin 1 and 77 Sabin 3 isolates were found among these samples at usual frequencies. Thus, sewage monitoring is the best way to screen for background circulation of discontinued Sabin viruses.

Sewage monitoring also has theoretical and practical limitations. In Moscow, there are about 200,000 children born per year and according to the vaccination schedule, each has received several doses of live OPV, meaning that millions of Sabin strains could have been excreted in Moscow over 10 years. Only 499 of these were identified by sewage monitoring. Therefore, this approach in its current state is theoretically incapable of detecting low-level circulation of a particular virus, including epidemically significant polioviruses (wild or VDPVs). It is likely that more viruses will be identifiable upon introduction of high throughput sequencing, but the survival rate of enteroviruses in sewage remains a major knowledge gap. In particular, it is not clear if the observed high year-to-year variation of isolation rates at the same facility is dependent on sampling methodology, sewage content, climate or some other factors. There have been attempts to correlate the number of poliovirus shedders to the concentration of poliovirus in sewage, but they were done on much smaller treatment facilities [29]. In large cities it may not be realistic to infer poliovirus circulation rates from sewage monitoring due to both lower detection rates and higher variance of virus prevalence, as observed in this study.

### 4.2. Sewage Monitoring of Enteroviruses

While poliovirus surveillance has a very clear objective of detecting wild and circulating VDPVs to aid the polio eradication campaign, the purpose of non-polio enterovirus surveillance is still being discussed. Sewage monitoring is also considered as a supplementary type of enterovirus surveillance. It is assumed that it has prognostic capabilities. That is, the emergence of a previously unknown type of virus in wastewater in the absence of manifest cases may indicate the beginning of its circulation among healthy population. However, significant differences in clinical incidence of infection by certain types and their frequency in sewage probes were found. There is no explicit evidence that enterovirus types are equally stable in sewage, especially as other contaminants and detergents can vary over different collection points and seasons. Moreover, there are examples of uneven stability. Echovirus 6 was the most frequent type isolated in sewage monitoring in Tunisia, and it was implied that E6 is more resistant to sewage treatment than other types [30]. Moreover, in our study, it was the type that featured a fair correlation between disease incidence and detection in sewage. Even different genotypes of a same type, such as E11, could be differently represented in sewage and among patients with overt disease [31]. It is not entirely clear if the observed differences in detection rates between sewage and incidence-based surveillance represent virus prevalence in the population, levels of the fecal excretion, virus stability in sewage, or efficiency of enrichment and isolation. In any case, it is obvious (and best exemplified by E30 incidence in AM cases and wastewater) that the sewage monitoring is not representative of clinical disease incidence caused by distinct EV types, in spite of the fact that in our research the same methodical approach was used for cell culture isolation of the viruses from wastewater and AM patients.

### 4.3. Methodology and Technical Efficiency.

Utility and efficiency of sewage monitoring relies upon many factors, including general sanitary, climate conditions, sampling and concentrating methods, sampling points, and screening methodology. The virus isolation rates in Moscow (mean over 10 years ranged from 14.4 to 34.6% at different facilities, 20.0% on average in the city) were comparable with other studies that used two-phase concentration (mixing of clarified wastewater with two polymers (dextran and polyethylene glycol) and subsequent settling, detailed in the WHO guidelines) and cell culture isolation: 22% and 30.1% in Italy [32,33], 25% in Greece [34]. Higher virus detection rates were observed at low-throughput facilities TP3 (34.6%) and TP4 (32.6%) in Moscow. As virus isolation was done in the same laboratory using the same protocol, this confirms that detection rates may be lower at larger facilities, which may be attributed to a higher degree of virus dilution. Detection rates at high-throughput facilities were still appropriate for monitoring, but the number of isolated viruses per million of potential virus shedding events was relatively lower here than in other studies. Moreover, a relatively low proportion of identified virus mixtures suggests that the method was operating at the sensitivity limit, while detection of only cytopathic types (Table 2) highlights a significant limitation of cell culture-based methods. Molecular detection and efficient concentration protocols provide much higher detection rates, which could be further enhanced by high-throughput sequencing for virus identification. The ratio of positive samples using molecular methods was 78.7% in Italy [35], 50–100% in Spain [36], 66–77% in Poland [37], 92.5% in Scotland [38], and 100% in France [39]. The repertoire of detected viruses is also much wider, up to 85–86 types [40,41] compared to just 31 types in this study. Using NGS for EV/PV surveillance requires some kind of enrichment, and different methods have distinct benefits. Physical enrichment of particles containing RNA protected by non-enveloped capsids yields the highest spectrum of viruses [42,43], but detects predominantly plant viruses and all picornaviruses may comprise just 3%–4% of total sequencing reads [42]. Cell culture enrichment using modified WHO protocols [44] has inevitable drawbacks of classical cell culture isolation and is well suited for detection of poliovirus, but not for non-cytopathic NPEVs. Virus particle immunoprecipitation might further enhance sensitivity to poliovirus [44] but would be much harder to implement for NPEV surveillance. The most promising approach to nucleic acid enrichment is probably PCR amplification [40,41]. Degenerate primers to capsid-encoding regions may vary in efficiency of amplification of distinct types, therefore using primers to conserved regions of 5′NTR and 2C genome regions [41] appears more robust, at least theoretically.

### 4.4. Other Findings

In Moscow, EVs were most frequently identified in the summer–autumn period right up to December with a maximum in September. Moreover, the NPEV predominates from June to October, and the PV in February to May. Since vaccination against poliomyelitis with OPV is carried out in Russia in accordance with the National Vaccination Schedule throughout the year, which excludes the effect of vaccination campaigns on the frequency of EV release, this suggests that the Moscow region is characterized by pronounced summer–autumn seasonality of NPEV circulation among the population. The high prevalence of the EV-B species among the NPEV viruses is consistent with the results of studies carried out in Europe [31,32,33,34,37,45] and seems to reflect the epidemiology of NPEV in the region. Viruses E7, E11 and E19 were isolated mainly from wastewater, which indicates the silent circulation of these types in the population and their low pathogenicity. On the contrary, for E30 we found a mismatch between the virus isolation from sewage and from cases of AM, which apparently indicates a high epidemic potential of the E30 virus for which the “silent” circulation is not typical.

### 4.5. Cost-Efficiency

An important but outstanding issue is the cost of enterovirus monitoring in wastewater. So far, the obvious advantage of sewage surveillance is that its organization is quite simple. It requires interaction with administration and engineering staff of a few treatment plants (rather than multiple hospitals) and minimal training of personnel for sampling and concentrating of samples (as opposed to implementation of meticulous AFP reporting network). Since the algorithm for examining samples in the laboratory does not differ in principle from the algorithm for examining patient stool samples, such surveillance can be easily organized in addition to clinical/AFP surveillance.

Precise costs of surveillance are hard to analyze since they come from several sources, such as core and project funding of several ministries and government offices. We have tried to summarize the relative costs of patient-based and environmental surveillance in Table 3. Environmental surveillance is much more efficient for monitoring silent virus circulation, such as VDPV introduction into the IPV-immunized population. However, AFP surveillance is more efficient (see above) for detection of virulent wild PV circulation or PV introduction after hypothetical IPV withdrawal in the distant future.

## 5. Conclusions

Virological studies of wastewater can provide important additional information about the circulation of PVs and NPEVs among the population, but their effectiveness as additional types of surveillance significantly depends on the methodology of monitoring, in which each component of the surveillance system matters, including the choice of the object, method of concentration, protocols of detection and identification of viruses. Cell culture-based assays detect only cytopathogenic viruses and do not provide a complete picture of the epidemic process of enterovirus infections. Therefore, improving the methodology of environmental investigation at all stages of the study is the most important task for improving the quality of surveillance for polio and enterovirus circulation and obtaining information about new NPEVs.

## Figures and Tables

**Table 1:** Enterovirus isolation frequency at Moscow sewage treatment plants (TP), 2004–2017.

TP	Year														
	2004	2005	2006	2007	2008	2009	2010	2011	2012	2013	2014	2015	2016	2017	2004–2017
1	44/ 159 **27.7**	39/ 195 **20.0**	30/ 146 **20.5**	25/ 153 **16.3**	17/ 147 **11.6**	14/ 143 **9.8**	15/ 132 **11.4**	28/ 136 **20.6**	30/ 154 **19.5**	27/ 139 **19.4**	24/ 133 **18.0**	27/ 109 **24.8**	23/ 254 **9.1**	49/ 153 **32.0**	392/ 2153 **18.2**
2	16/ 159 **10.1**	28/ 156 **17.9**	17/ 153 **11.1**	23/ 159 **14.5**	16/ 153 **10.5**	21/ 156 **13.5**	36/ 150 **24.0**	19/ 153 **12.4**	29/ 153 **19.0**	30/ 153 **19.6**	18/ 141 **12.8**	18/ 150 **12.0**	12/ 156 **7.7**	27/ 156 **17.3**	310/ 2148 **14.4**
3	24/ 36 **66.7**	4/ 22 **18.2**	21/ 40 **52.5**	11/ 50 **22.0**	25/ 48 **52.1**	24/ 51 **47.1**	26/ 52 **50.0**	27/ 51 **52.9**	25/ 45 **55.6**	10/ 50 **20.0**	3/ 48 **6.3**	12/ 50 **24.0**	5/ 57 **8.8**	4/ 39 **10.3**	221/ 639 **34.6**
4	5/ 8 **62.5**	4/ 18 **22.2**	4/ 18 **22.2**	16/ 38 **42.1**	13/ 34 **38.2**	17/ 44 **38.6**	25/ 41 **61.0**	24/ 42 **57.1**	21/ 43 **48.8**	12/ 42 **28.6**	7/ 39 **17.9**	4/ 50 **8.0**	6/ 49 **12.2**	8/ 44 **18.2**	166/ 510 **32.5**
Total	89/ 362 **24.6**	75/ 391 **19.2**	72/ 357 **20.2**	75/ 400 **18.8**	71/ 382 **18.6**	76/ 394 **19.3**	102/ 375 **27.2**	98/ 382 **25.7**	105/ 395 **26.6**	79/ 384 **20.6**	52/ 361 **14.4**	61/ 359 **17.0**	46/ 516 **8.9**	88/ 392 **22.4**	1089/ 5450 **20.0**

Numerator—the number of positive samples; denominator—the number of samples studied; bold—% positive samples.

**Table 2:** Isolation of non-polio enteroviruses isolated from sewage, Moscow, 2004–2017.

		Year														
Species	Type	2004	2005	2006	2007	2008	2009	2010	2011	2012	2013	2014	2015	2016	2017	2004–2017
EV-A n = 28	CVA3								1	1	1					3
	CVA7					6	2	4					1			13
	CVA10										1	1				2
	CVA16					1			4		3		1		1	10
EV-B n = 556	CVB1											1	1	1		3
	CVB2				1											1
	CVB3				2	2		2	8	3					2	19
	CVB4						1	3	1	2	1	2	1		5	16
	CVB5			2	2		1			9	5	1	3		2	25
	CVB6						1				1					2
	E1	1	1													2
	E2	1														1
	E3	20	5		3	3	3	3	3			2				42
	E4		2				3				1			1		7
	E6	8	3	9	4	1	1	7	14	12	1	7	1	2		70
	E7	13	12	10	20	5	23	21	9	11	11	2	9	4	3	153
	E11	9	3	5	3	17	9	9	12	9	16	8	6	2	5	113
	E12		1	1	2		5		1				1		2	13
	E13	1	1	1					1			1		1		6
	E14	1	1									1				3
	E17					1		3								4
	E19					5	5	9	2	3			1			25
	E20	1	1	1				1	4							8
	E25	2	1	3	5			3	1		1					16
	E29	2	1	2	2		1		2	4						14
	E30		1	1	2	2		1			1	2				10
	E33		1	1												2
	EV31													1		1
EV-C n = 11	CVA17							1	1							2
	CVA21										1					1
	CVA24						1		1	3	3					8
Total	2004–2017	59	34	36	46	43	56	67	65	57	47	28	25	12	20	595

**Table 3:** Relative cost and efficiency of poliovirus and enterovirus surveillance.

	Patient-Based	Environmental
Cost per sample	About equal. Higher material costs of sewage sampling are balanced by indirect costs of AFP reporting.	
Isolation efficiency per sample tested	3%: 8 polio isolates from 250 AFP cases screened	9%: 499 isolates from 5450 samples tested in 2004–2017
Isolation efficiency per population	0.05 isolates/million persons/year	3 isolates/million persons/year
